# Oral PrEP for young African women and men

**DOI:** 10.7448/IAS.19.1.20780

**Published:** 2016-02-17

**Authors:** Roberto Parulan Santos

**Affiliations:** Pediatrics, Bernard & Millie Duker Children's Hospital, Albany Medical Center Albany, NY, USA

In the manuscript by Celum *et al*. [1], the rationale and principles for “rethinking HIV prevention to prepare for oral pre-exposure prophylaxis (PrEP) implementation for young African women” were thoroughly discussed. The authors listed several reasons for the urgent need to add pre-exposure prophylaxis or PrEP to the growing body of effective HIV-prevention strategies among young women aged below 25 years, a high-risk population. This age group remains one of the most disproportionately affected groups by incident HIV infections worldwide [2]; however, certain aspects of their commentary need clarification and further review.

First, they noted that young African women will benefit from PrEP assuming they remain HIV negative. As mentioned in the manuscript [1], women aged below 25 years and living with HIV constitute approximately 75% (three out of four million) of young people in sub-Saharan Africa. I agree with providing PrEP to young African women to prevent HIV infection and this should be extended to young pregnant women in an effort to prevent mother-to-child transmission in serodiscordant couples [3]. It would be of interest if the authors consider implementation of PrEP among pregnant young women since mother-to-child HIV transmission rates remain high in sub-Saharan Africa [4].

Second, in every sexual relationship, one other sexual partner is involved who should be included in the prevention programme. Lest we forget, it takes two to tango. If the sexual partner is HIV positive, then combination antiretroviral therapy (cART) should be offered consistent with the principle “treatment as prevention or TasP” [3]. Transmission of HIV may not be zero in serodiscordant couples; however; it is decreased when HIV-positive partners are on cART together with counselling on harm reduction and provision for condoms [5]. By extension, are there programmes in the pipeline locally addressing oral PrEP implementation for young African men if they remain HIV negative?

Third, given the expense associated with PrEP, cost-effectiveness in *resource-limited* settings should be addressed prior to implementation. In *resource-sufficient* settings, PrEP for adolescents and young adults rely on both private and public medical insurance programmes [2] not only for the supply of PrEP regimen (TDF/FTC) but also for linkage and retention to medical care. Well-funded research environment may be different in real-world setting in sub-Saharan Africa, and the authors should comment on sustainability and durability of PrEP among young African women to optimize uptake and maximize effective use of this first generation biomedical HIV prevention product.

In conclusion, the article by Celum *et al*. describes an important prevention platform with the use of PrEP among young African women that requires clarification and further review prior to implementation in resource-limited settings as well as extending the programme to young pregnant women and men in sub-Saharan Africa.

## Author information

RPS is a member of the American Academy of HIV Medicine (AAHIVM) New York/New Jersey Steering Committee; a member of the Perinatal Transmission Committee, New York State – Department of Health; and a peer reviewer for HIV Clinical Resource – Online Guideline for the New York State Department of Health on “Care of the HIV-Exposed Infant with Indeterminate Status,” published online in January 2014.
